# Evaluation of In-Cloth versus On-Skin Sensors for Measuring Trunk and Upper Arm Postures and Movements

**DOI:** 10.3390/s23083969

**Published:** 2023-04-13

**Authors:** Damien Hoareau, Xuelong Fan, Farhad Abtahi, Liyun Yang

**Affiliations:** 1 Department of Mechatronics, École Normale Supérieure de Rennes, 35170 Bruz, France; 2 Laboratoire SATIE, CNRS UMR 8029, École Normale Supérieure de Rennes, 35170 Bruz, France; 3 Institute of Environmental Medicine, Karolinska Institutet, Solnavägen 4, SE-171 77 Stockholm, Sweden; 4 Division of Ergonomics, School of Engineering Sciences in Chemistry, Biotechnology and Health, KTH Royal Institute of Technology, Hälsovägen 11C, SE-141 57 Huddinge, Sweden; 5 Department of Clinical Science, Intervention and Technology, Karolinska Institutet, SE-171 77 Stockholm, Sweden; 6 Department of Clinical Physiology, Karolinska University Hospital, SE-141 57 Huddinge, Sweden

**Keywords:** wearable sensors, physical ergonomics, inertial measurement units, risk assessment, accuracy, smart workwear systems, musculoskeletal disorders

## Abstract

Smart workwear systems with embedded inertial measurement unit sensors are developed for convenient ergonomic risk assessment of occupational activities. However, its measurement accuracy can be affected by potential cloth artifacts, which have not been previously assessed. Therefore, it is crucial to evaluate the accuracy of sensors placed in the workwear systems for research and practice purposes. This study aimed to compare in-cloth and on-skin sensors for assessing upper arms and trunk postures and movements, with the on-skin sensors as the reference. Five simulated work tasks were performed by twelve subjects (seven women and five men). Results showed that the mean (±SD) absolute cloth–skin sensor differences of the median dominant arm elevation angle ranged between 1.2° (±1.4) and 4.1° (±3.5). For the median trunk flexion angle, the mean absolute cloth–skin sensor differences ranged between 2.7° (±1.7) and 3.7° (±3.9). Larger errors were observed for the 90th and 95th percentiles of inclination angles and inclination velocities. The performance depended on the tasks and was affected by individual factors, such as the fit of the clothes. Potential error compensation algorithms need to be investigated in future work. In conclusion, in-cloth sensors showed acceptable accuracy for measuring upper arm and trunk postures and movements on a group level. Considering the balance of accuracy, comfort, and usability, such a system can potentially be a practical tool for ergonomic assessment for researchers and practitioners.

## 1. Introduction

Work-related musculoskeletal disorders (MSDs) remain a substantial burden to individuals, organizations, and societies worldwide. In Europe, MSDs are the most prevalent work-related health problem: about 43% of European Union (EU) workers reported back pain, and 41% reported muscular pains in the shoulders, neck, and/or upper limbs in 2015 [1]. Work in tiring positions is still common in current workplaces, as reported by 43% of workers for being exposed to at least a quarter of their work time in the EU [1]. In Sweden, it has been estimated that the total costs of MSDs were 102.3 billion SEK in 2012, which equaled 2.8% of the national gross domestic product (GDP) [2].

In order to design effective intervention programs and prevent MSDs, a better understanding of the underlying mechanisms between exposures and outcomes, the development of practical and reliable risk assessment methods, and a wider use of such high-quality risk assessment methods are among the key steps as suggested by researchers [3,4,5]. However, physical exposure has generally been assessed via questionnaires [6], which suffer from low accuracy and bias and lack detailed information on exposure frequency or intensity [7,8]. Exposure assessed with observational methods can also suffer from being sampled for a relatively short period of the workday and high inter-rater variability [9,10]. A limited number of studies included physical exposure data based on direct measurement and its association with occupational health outcomes. Two recent studies showed that direct measured arm elevation and trunk forward bending have a dose-response association with long-term sickness absence [11,12]. With directly measured data of high accuracy, researchers found that ten more minutes of work time with the arm elevated more than 60° was associated with approximately 50% higher risk of long-term sickness absence in four years, and 5 more minutes of work time with backward bending over 60° was associated with 8% higher risk [11,12]. In addition, an action level for the median arm velocity has been proposed for the prevention of MSDs in the neck and upper extremities [13].

The latest technical development in wearable technology has provided opportunities to perform ergonomic risk assessments and interventions with accurate and convenient methods. Thanks to the growing market for wearable sensors, the development of new technologies is increasing for the risk assessment of work-related musculoskeletal disorders [14]. Systems composed of body area sensor networks providing continuous and automatic measurement have been created [15,16]. Smart workwear systems allowing ergonomic risk assessment are emerging and are being commercialized. Wergonic AB, Stockholm, Sweden (wergonic.se) offers a solution to monitor the ergonomic risk using a cloth with embedded sensors. Sensors are placed into pockets at three locations, i.e., both upper arms and the upper back. The data collection is performed using wireless communication with a smartphone, and real-time feedback can be provided based on preset thresholds.

The inertial measurement unit (IMU) is a widely used type of sensor for motion- and posture-related applications, with benefits of high accuracy, ease of implementation, and low user burden [17,18,19,20]. A recent literature review on wearable inertial sensors for human motion analysis showed the increasing applications of such wearable sensors in industrial settings due to their portability, low cost, minimal invasiveness, and applicability outside of the laboratory environment [21]. More than half of the identified systems also provide real-time data analysis, which is an advantage for industrial applications including risk assessment, motion tracking to assist the design of collaborative robotics, and human action recognition [21]. Nevertheless, the information provided by IMUs is related to their placement and fixation. Cloth-embedded sensors face relative motion artifacts, which can impact the measurement quality [22,23]. Improvements can be obtained by using tight-fitting clothing, and good agreement between skin-mounted and cloth-embedded sensors has been shown for temporal motion kinematics at C7 and T12 locations [24]. Moreover, other external factors may impact the measurements, such as sensor fixation or soft tissue artifacts [25].

Depending on the application, several methods are reported in the literature to compensate for and evaluate the cloth artifacts. Previous work presented artificial intelligence-based algorithms for assessing errors between sensors in a loose garment and an optical tracking system [26]. However, in order to compensate for these potential errors in a smart workwear system, they must first be examined and quantified. To the best of our knowledge, few studies have investigated the impact of IMU sensors embedded in cloth and used in pockets for measuring trunk and upper arm postures and movements.

Hence, this study aimed to evaluate the performance of in-cloth sensors compared to on-skin sensors for measuring trunk and upper arm postures and movements during simulated occupational activities. Commonly used ergonomic exposure parameters, including the upper arm and trunk inclination angles, two types of upper arm velocities (the inclination velocity and the generalized velocity), and trunk inclination velocities, were calculated and compared for each occupational activity. and the resulting differences from the comparisons can provide knowledge about the accuracy and limitations of measurements for the practical use of smart workwear systems both in the lab and in the field.

## 2. Materials and Methods

### 2.1. Demographic Data

Twelve volunteers (five males and seven females) were involved in this study. Before the experience, they were informed about the study and signed informed consent. The mean (±standard deviation) age of the participants was 32.8 ± 11.3 years, the height was 174.2 ± 10.2 cm, the weight was 68.7 ± 10.2 kg, and the BMI was 22.6 ± 2.7 kg/$m^{2}$. Eleven participants are right-handed, and one is left-handed. The study was approved by the Regional Ethics Committee in Stockholm (Dnr: 2019-01206).

### 2.2. Experimental Setups

**Figure 1:**
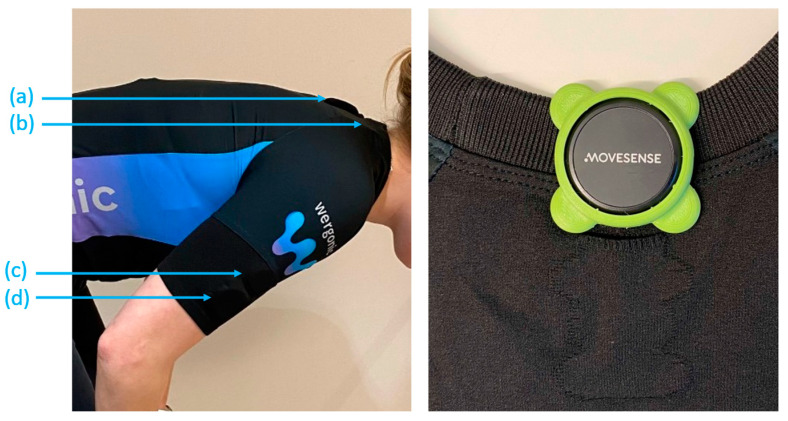
To the left: the two sensor setups showing (**a**) the trunk sensor in the shirt; (**b**) the trunk sensor on the skin; (**c**) the right upper arm sensor on the skin; and (**d**) the right upper arm sensor in the shirt. To the right: the Wergonic T-shirt pocket and the matching sensor case.

For this study, two sets of inertial measurement units were used (Figure 1), with each set containing three sensors (Movesense, Suunto, and Helsinki, Finland). The first set of sensors was attached directly to the skin using double-sided tape, with two on the upper arms at the insertion of deltoids and one on the upper back at the level of T1–T2 vertebrae. An additional piece of medical tape was put above the sensors on the skin to avoid relative movement. This setup is referred to as “skin sensors” in the following text.

The second set of sensors was placed in an elastic T-shirt (Wergonic AB, Stockholm, Sweden), with pockets placed at both the upper arms and upper back for the IMU sensors. The shape of the pocket and the extra sensor case with a matching shape feature were designed to prevent sensor rotation and limit relative movement errors (Figure 1). The second setup is referred to as “cloth sensors” in the following text. The shirt size, with a range of small to extra-large, was chosen for each participant to be comfortable and tight. The two sets of sensors were placed close to each other without overlapping.

Both the accelerometer and the gyroscope data from the IMU sensors were sampled at 104 Hz and collected by the Movesense showcase iPhone application (Amer Sports Digital Services Oy, Helsinki, Finland) using Bluetooth.

### 2.3. Experimental Protocol

**Figure 2:**
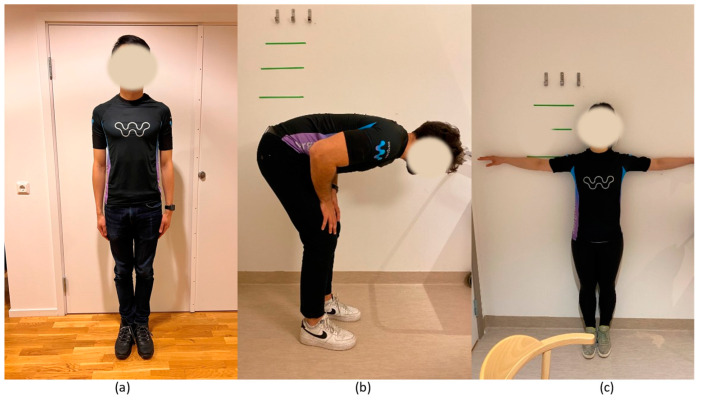
Calibration poses: (**a**) I-pose of standing straight with arms relaxed by the body; (**b**) forward trunk bending at about 90 degrees; and (**c**) T-pose of standing straight with both arms lifted at about 90 degrees.

The experiment consisted of calibration steps and simulated work tasks. The calibration was necessary for the data fusion presented in the next section. It consisted of three calibration poses, and participants were instructed to hold each pose still for three seconds (Figure 2):

(a) I-pose: stand up straight and look straight forward with arms at each side;
(b) Forward trunk bending: bow forward at about 90°;
(c) T-pose: stand up straight and look straight forward, and hold the arms horizontally to the sides at 90°.

**Figure 3:**
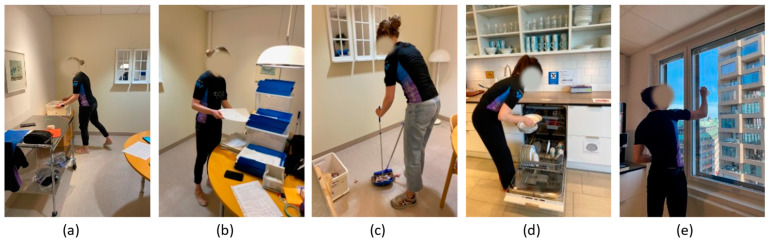
Performed simulated work tasks: (**a**) Lifting boxes; (**b**) sorting mail; (**c**) wiping floor; (**d**) cleaning dishwasher; and (**e**) cleaning windows.

After the calibration, participants were introduced to the work tasks and instructed to perform the tasks as they would naturally do. When possible, they were also instructed to use their dominant hand to mainly perform the tasks. The duration of each task was two minutes. The different tasks were chosen to represent work scenarios using the upper arms and back at low and high angle amplitudes and velocities. This allows the assessment of the shirt setup in different conditions of use. The tasks performed were as follows (Figure 3):

(a) Lifting boxes: lift a light box from the floor to the table in front and put it back, and from the floor to the table to the side and put it back;
(b) Sorting mail: sort mail with marked letters into the corresponding compartments at different heights;
(c) Wiping floor: clean up paper scraps on the floor and put them into a box using a shovel and broom;
(d) Cleaning dishwasher: empty cups and plates from the dishwasher and store them on shelves;
(e) Cleaning windows: clean windows with markers at different heights using a rag and spray bottle.

### 2.4. Data Fusion and Signal Processing

Raw data from the IMUs were processed in MATLAB (version R2022a, MathWorks Inc., Natick, MA, USA). The inclination angle, inclination velocity, and generalized velocity were computed for the sensors on the arms. The sagittal inclination angle and sagittal inclination velocity were computed for the trunk. The posture and movement computations of both the arms and trunk followed the processing steps described in Fan et al. [27]. Firstly, data from accelerometers and gyroscopes were integrated with a sensor fusion algorithm to reduce the effects of non-gravitational (dynamic) acceleration and generate corrected gravitational acceleration. In the sensor fusion algorithm, the original data were resampled to 128 Hz and processed by a Kalman filter with the recommended coefficients [28]: 0.005 rad/s for the gyroscope white noise, 0.1 $m/s^{2}$ for the accelerometer white noise, and 0.0005 $\mathrm{rad}/s^{2}$ for the gyroscope bias. Then, the corresponding angles of each body part were calculated using the reference poses:

- Inclination angles (arms): upper arm inclination angles were obtained by calculating the relative angle to the reference I-pose [29];
- Forward/Sagittal inclination angles (trunk): the forward inclination angles (inclination angles on the sagittal plane) were obtained using Hansson forward/backward projections, the corresponding I-pose as the reference, and forward trunk bending to indicate the direction [30].

Synchronization between the two sets of sensors was performed using cross-correlation and then visually checked for each individual participant. Finally, two types of angular velocities were calculated for comparison since both computational methods had been used and reported in previous research [31,32,33,34]. In addition, recent studies have identified large differences in the values between these two computational methods [27,29,35]. Since there are currently no standard metrics for assessing the arm’s angular velocity, the performance of the in-cloth sensors vs. on-skin sensors using both metrics is worth evaluating. The two types of angular velocities were described below:

- The inclination velocities (arms and trunk): were computed by using a simple temporal derivation, i.e., dividing the difference between two samples of inclination angles by the sampling time;
- The generalized velocities (arms): the upper arm generalized velocities were obtained [30] by dividing the angular difference of the gravitation vectors between two samples on a unit sphere with the sampling time [30,35].

### 2.5. Statistical Analysis

After synchronizing and extracting the upper arm and trunk angles and velocities of each work task, a comparison between the skin sensors and cloth sensors was made on the following parameters: For the upper arm and trunk inclination angles, the 5th, 10th, 50th, 90th, and 95th percentiles of the angles and the percentage of time with the angles less than 20°, as well as the time over 30°, 45°, 60°, and 90°, were calculated. For the upper arm inclination and generalized velocities, as well as the trunk inclination velocities, the 5th, 10th, 50th, 90th, and 95th percentiles were calculated. A paired comparison was made by using the mean absolute error (MAE) and its standard deviation (SD) for all parameters for each work task. In addition, Bland–Altman plots of the median and the 90th percentile of the upper arm and trunk angles and inclination velocities for all tasks were applied to show the differences and the limits of agreement (calculated as mean ± 1.96 SD) between the two sensor setups.

## 3. Results

### 3.1. Angular Distributions

For the dominant upper arm, the cloth-sensor setup generally had small MAEs compared to the skin-sensor setup, ranging from 1.2° to 4.1° for the median upper arm inclination angle (Table 1). Larger errors were observed for the cleaning dishwashers and cleaning windows tasks when looking at the higher percentiles, with MAEs of 7.6° and 7° for the 90th percentile angle and MAEs of 8.3° and 7.4° for the 95th percentile angle. The differences were smaller in the non-dominant upper arm, with the MAE ranging from 1.3° to 2° for the median upper arm inclination (Table A1 in the Appendix).

**Figure 4:**
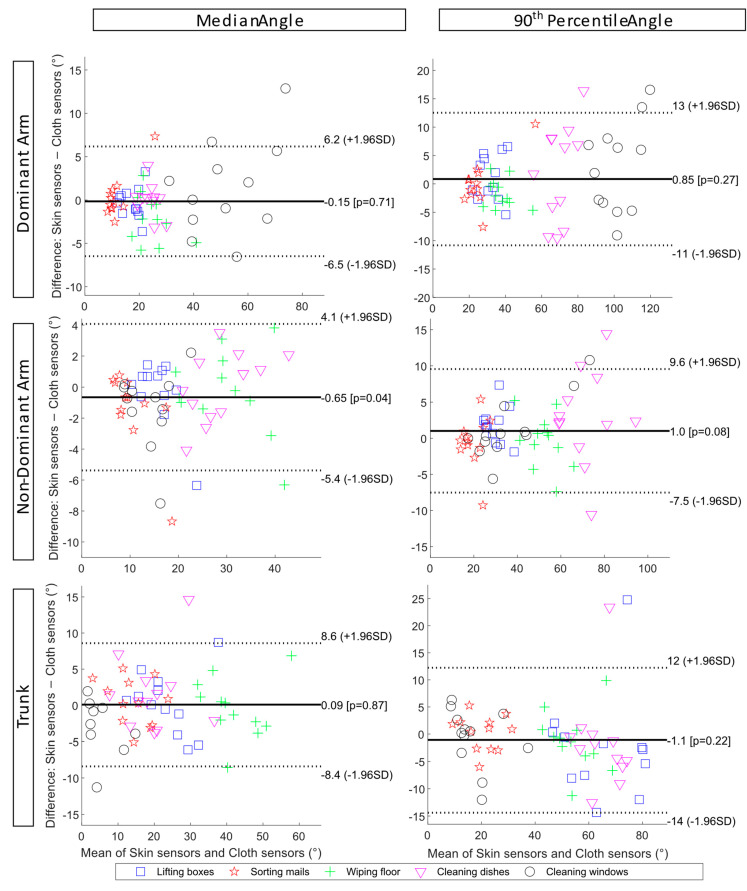
Bland–Altman plots of the upper arm inclination and trunk forward inclination angle during the five simulated work tasks show the limits of agreements between skin sensors and cloth sensors. From top to bottom: the dominant arm, the non-dominant arm, and the trunk. To the left are the median angles, and to the right are the 90th percentile angles.

The differences and limits of agreement between the skin sensors and cloth sensors during the simulated tasks for the dominant and non-dominant arms are also presented with Bland–Altman plots in Figure 4. Similarly, larger differences were observed for the cleaning dishwasher and cleaning windows tasks. For the dominant arm, the mean difference was −0.15° for the median inclination angle, and the limits of agreement were −6.5° and 6.2°. The mean difference for the 90th percentile dominant arm inclination was 0.85°, with limits of agreement of −11° and 13°. For the non-dominant arm, the limits of agreement were smaller than those for the dominant arm, with −5.4° and 4.1° for the median inclination angle and −7.5° and 9.6° for the 90th percentile inclination angle.

**Figure 5:**
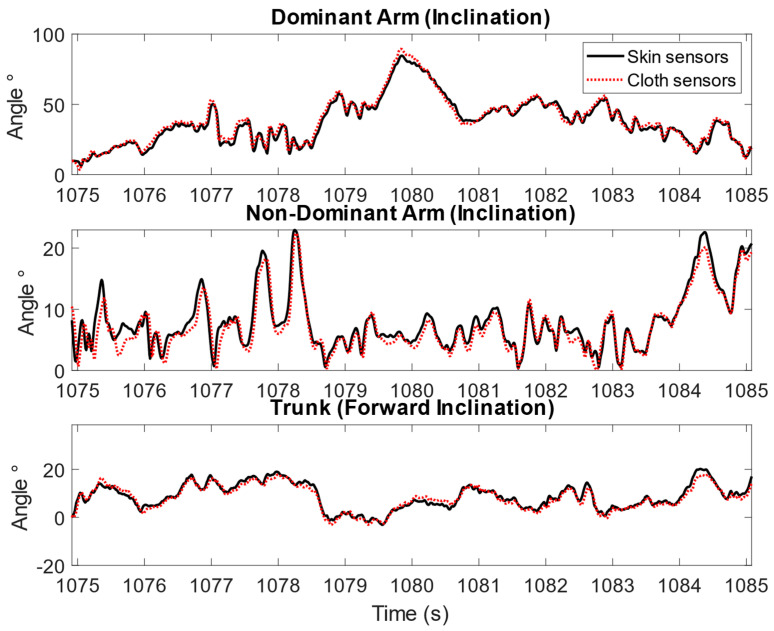
An example of the upper arm inclination and trunk forward inclination angles measured by skin sensors and cloth sensors for one participant during the simulated window cleaning task for 10 s, showing good agreement between the skin sensors and cloth sensors.

**Figure 6:**
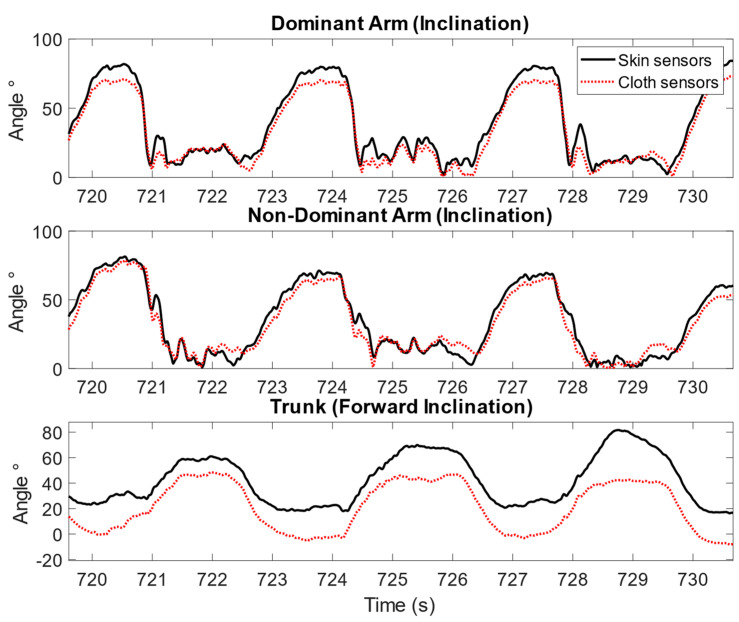
An example of the upper arm inclination and trunk forward inclination angles measured by skin sensors and cloth sensors for one participant during the simulated dishwasher cleaning task for 10 s, showing worse agreement between the skin sensors and cloth sensors.

In addition, individual differences were observed, and larger errors between the cloth sensors and skin sensors were observed for a few participants. Figure 5 and Figure 6 illustrate this variance in the time-series angular measurements of the cloth sensors against the skin sensors. In Figure 5, the angular measurements by the cloth sensors were in good agreement with the skin sensors, as illustrated by the example of one participant cleaning windows. As a comparison, in Figure 6, larger differences were observed, as shown by the example of one participant cleaning the dishwasher. The differences became larger when the arms were lifted higher for the upper arms, and a constant difference was observed for the trunk inclination throughout the task.

For the trunk, the MAEs between the cloth and skin sensors ranged from 2.7° to 3.7° for the median forward inclination angle (Table 2). The maximum MAEs were observed for the lifting boxes and cleaning dishwasher tasks, with MAEs equal to 6.8° and 5.8° for the 95th percentile angles, respectively. For the percentage of time spent with angles less than 20°, the largest difference was observed for the task of sorting mail, with the MAE equal to 10.7%. A potential reason could be that during this specific task, the participants spent a lot of time around 20° trunk inclination (mean time percentage of 78%), and the error would lead to misclassification for trunk inclination <20°.

The Bland–Altman plots show the limits of agreement between the skin sensors and cloth sensors for the trunk inclination angle (bottom row, Figure 4). The mean difference of the median trunk inclination was 0.09°, with limits of agreement of −8.4° and 8.6°. Larger differences are observed for the 90th percentile trunk inclination, with a mean difference of −1.1° and limits of agreement of −14° and 12°. In addition, individual differences were observed, especially during the tasks of lifting boxes and cleaning the dishwasher.

### 3.2. Angular Velocity

For the dominant arm, the MAEs between the cloth and skin sensors were generally small, ranging from 1°/s to 4.5°/s for the median inclination velocity (Table 3). Maximum errors are found for the sorting mail and cleaning windows tasks, with MAEs equal to 15.3°/s and 26.1°/s for the 95th percentile inclination velocity, respectively. These larger differences might be due to the sleeves not following the upper arm movements properly, especially during faster motions and at high inclination angles. For the non-dominant arm, the MAEs between the two sensor setups of the median inclination velocity ranged from 0.5°/s to 2.1°/s (Table A2 in the Appendix). The MAEs of the median trunk forward inclination velocity had smaller values, ranging from 0.4°/s to 2°/s (Table 4). The lifting boxes task had the largest difference, with MAE equal to 13.2°/s for the 95th percentile inclination velocity.

**Figure 7:**
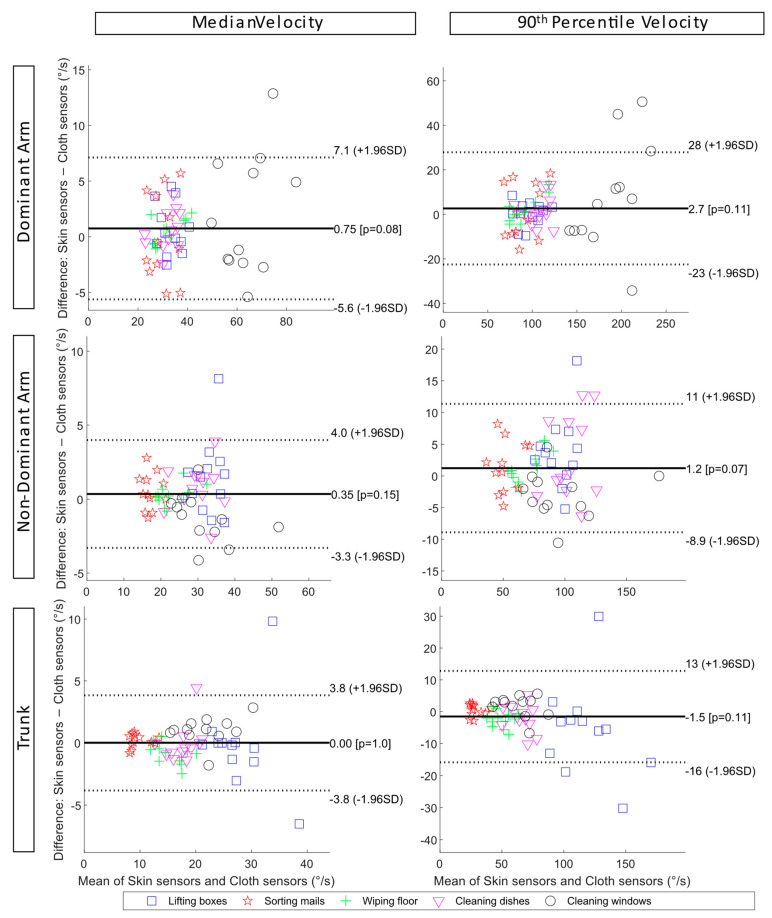
Bland–Altman plots of the upper arm inclination velocity and trunk forward inclination velocity during the five simulated work tasks show the limits of agreements between skin sensors and cloth sensors. From top to bottom: the dominant arm, the non-dominant arm, and the trunk. To the left is the median inclination velocity, and to the right is the 90th percentile inclination velocity.

The limits of agreement between the skin sensors and cloth sensors of the upper arms and trunk inclination velocities during the simulated tasks are also shown as Bland–Altman plots in Figure 7. For the dominant arm, the mean difference value was 0.75°/s, and the limits of agreement were −5.6°/s and 7.1°/s for the median inclination velocity. The larger dispersion of data points was observed for the window cleaning task. This could be partly due to the large variance in individual work techniques. For the 90th percentile inclination velocity of the dominant arm, the mean difference value was 2.7°/s, and the limits of agreement were −23°/s and 28°/s. For the trunk median inclination velocity, the mean difference was 0°/s, and the limits of agreement were −3.8°/s and 3.8°/s. For the 90th percentile trunk inclination velocity, the mean difference value was −1.5°/s, and the limits of agreement were −16°/s and 13°/s. A larger dispersion was observed for the box-lifting task.

The generalized angular velocities showed significantly higher differences between the two sensor setups. For the median upper arm generalized velocity, compared to the upper arm inclination velocity, the maximum MAEs increased from 3.8°/s to 15.3°/s for the dominant arm and from 2.3°/s to 3.9°/s for the non-dominant arm (Table A3 and Table A4). The differences became more evident when looking at the 95th percentile of angular velocity. This could be explained by the definition of generalized angular velocity, where movements in all directions are included, compared to inclination velocity, where the only change in inclination is included.

## 4. Discussion

This study evaluated in-cloth against on-skin sensors for measuring trunk and upper arm postures and movements for smart workwear systems during simulated work tasks. For most tasks, high agreements between the two sensor setups were observed for the upper arm and trunk angles. For the arm, slightly higher errors were observed for the 90th and 95th percentiles of arm inclination angle and velocity during cleaning windows and cleaning the dishwasher. For the trunk, slightly higher errors were observed for the 90th and 95th percentiles of trunk inclination and velocity for lifting boxes and cleaning the dishwasher. The generalized velocity had distinctively higher errors for both the upper arms and trunk. The in-cloth sensors showed acceptable accuracy on a group level for measuring upper arm and trunk inclinations and inclination velocities.

The simulated tasks in this study were chosen to cover a large range of work activities that may involve arm and trunk movements, thus evaluating the in-cloth sensors in different settings. Activities like cleaning windows and cleaning dishwashers involved higher movement amplitudes for the dominant arm. The errors of the in-cloth sensor compared to on-skin sensors were higher in these cases, which is to be expected. These larger differences might also be due to the sleeves not following the upper arm movements properly, especially during faster motions and at high inclination angles. As shown in Table 1, the MAEs increased in general from the 5th to the 95th percentile of the upper arm angle. Still, the MAEs were less than 4.1° for all the median arm inclination values. A similar phenomenon was observed in the arm inclination velocities (Table 3). The median arm inclination velocity had MAEs smaller than 4.5°/s in all tasks. Higher errors were observed when the generalized velocities were calculated (Table A3 in the Appendix). The maximum MAE for the median generalized velocity was 15.3°/s during window cleaning (the reference value was 124.2°/s), and the MAEs were significantly higher for the 95th percentile of arm generalized velocity. This is expected since the definition of generalized velocity includes motions on all planes, compared to inclination velocity, which only includes motions/changes in the inclination. Therefore, the performance of the in-cloth sensors can be affected to a greater degree by the cloth and motion artifacts during the tasks.

For the non-dominant arm, the in-cloth sensors had lower MAEs than the dominant arm regarding the inclination angle and velocity (Table A1). This was also expected as the non-dominant arm was less used. The maximum MAE was observed for the 95th percentile inclination angle while cleaning the dishwasher, during which participants usually used their non-dominant arm to a greater degree. For the median inclination angles, the MAEs were less than 2° for all tasks. Concerning the non-dominant arm inclination velocities (Table A2), the overall MAEs were smaller than 6.6°/s. Higher MAEs were also observed for the non-dominant arm generalized velocities (Table A4 in the Appendix A).

Regarding the trunk, lifting boxes and cleaning dishwashers involved higher movement amplitudes. The maximum MAE for trunk forward inclination angles was 6.8° for all tasks, which was observed during lifting boxes (Table 2). In general, the errors for trunk inclination velocity were quite small, with maximum MAEs of 2°/s and 13.2°/s for the median and 95th percentile values, respectively, observed during the lifting boxes task (Table 4).

One thing worth noticing is that the MAEs for trunk inclination remained on a similar level from the 5th percentile to the 95th percentile throughout each task, even when the trunk’s forward inclination angle was small. Whereas for the upper arms, the MAEs in general increased for the higher percentiles of arm inclination (Table 1) and when the arms were lifted higher. This type of error is further illustrated in Figure 6. The relatively constant error for the trunk could be caused by the non-optimal fit of the clothes. The looseness of the garment where the trunk sensor was located or a potential overlap of the cloth sensor and skin sensor could lead to the cloth sensor having a slightly different tilt compared to the skin. Regarding the errors observed for the upper arms, they could potentially be caused by the elasticity of the sleeve fabric, leading to slightly larger cloth artifacts when lifting the arms high.

In addition to the fit of the clothes, different individual work techniques and individual height may also imply variances in the level of errors. For example, there was a high variance in the individual arm inclination angles and velocities during cleaning windows and the dishwasher and a high variance in trunk velocities while lifting boxes. Therefore, this variance is good to include in the experiment so the results can represent different work scenarios and individuals.

Another limitation was the placement of the two sensor setups, which should ideally be at the same location, i.e., at the insertion of the deltoids and the level of T1–T2 vertebrae. However, since overlapping of the sensors was undesirable, they could not be placed in the same place. Therefore, the cloth sensors were placed carefully close to the skin sensors without overlapping each other. However, for a few participants, the overlapping of the cloth sensors on the skin sensors of the upper arms was observed. This can lead to overestimated errors of the cloth sensors since normal wear of the T-shirt will be tighter on the skin and potentially a better fit on the body without another sensor in between.

Future studies can look into error-correcting algorithms for the in-cloth sensors set up to improve their performance for smart workwear systems. This study highlights the existing errors in such a system and can contribute to how to find the most adapted approach in future studies. One potential method is the use of artificial intelligence-based algorithms; for example, Lorenz et al. [16] used a probabilistic neural network based on a supervised learning method to reduce loose cloth artifacts.

## 5. Conclusions

This work evaluated the in-cloth sensors against the on-skin sensors in simulated work tasks for upper arms and trunk posture assessment. Errors from in-cloth sensors were quite low for all median values of inclination angles and velocities. Larger errors were observed for the 90th and 95th percentiles of inclination angles and velocities. The performance depended on the tasks and was affected by individual factors, such as the fit of the clothes. Nevertheless, future work should compensate for the cloth artifacts and thus improve measurement accuracy. In conclusion, in-cloth sensors showed acceptable accuracy for measuring upper arm and trunk postures and movements on a group level. Considering the compromise between accuracy, comfort, and usability, such a system is potentially a practical tool for ergonomic assessment for researchers and practitioners.

## Figures and Tables

**Table 1:** The mean and the standard deviation (mean ± SD) of the mean absolute errors (MAEs) of the dominant upper arm inclination angle between cloth sensors and skin sensors during the five simulated tasks, with the reference value of skin sensors shown in brackets (*n* = 12).

Dominant Arm, Inclination	Simulated Work Tasks				
	Lifting Boxes	Sorting Mails	Wiping Floor	Cleaning Dishwasher	Cleaning Windows
Percentile (°)					
5th	0.7 ± 0.5 (4.5)	1 ± 1.8 (3.7)	2.2 ± 1.6 (8.4)	0.6 ± 0.4 (5.6)	1.7 ± 1.2 (10.2)
10th	0.7 ± 0.7 (6.4)	1.1 ± 1.8 (5)	2.4 ± 1.7 (11.4)	0.9 ± 0.3 (7.9)	2.5 ± 2.8 (15.8)
50th	1.4 ± 1.1 (17.1)	1.5 ± 1.9 (11.8)	2.7 ± 2 (23.6)	1.2 ± 1.4 (23.6)	4.1 ± 3.5 (52.7)
90th	3.5 ± 2.1 (32.4)	2.8 ± 3.1 (25.6)	2.5 ± 1.6 (36.6)	7.6 ± 3.8 (70.8)	7 ± 4.3 (103.2)
95th	4.1 ± 2.2 (36.2)	3.6 ± 3.3 (34.3)	2.2 ± 1.4 (40.8)	8.3 ± 3.8 (83.1)	7.4 ± 5 (112.2)
Percentage of time (%)					
<20°	6.1 ± 4.4 (61.8)	4.6 ± 5.8 (81.1)	9.3 ± 8.5 (39.3)	2.6 ± 2.8 (41.8)	2.2 ± 2 (18.9)
>30°	3.1 ± 2.7 (13.9)	2.6 ± 4.1 (8.3)	7.1 ± 6.1 (27.2)	2.5 ± 3.1 (38.8)	2.4 ± 1.3 (71.4)
>45°	1.6 ± 2.5 (1.7)	1.3 ± 2.5 (3.4)	2.1 ± 4 (4.7)	1.5 ± 2 (23.4)	3.7 ± 2.5 (57.8)
>60°	0.1 ± 0.2 (0.1)	0.6 ± 1 (1.2)	0.4 ± 1.1 (0.2)	3.3 ± 2.4 (15.8)	4.4 ± 3.6 (44.6)
>90°	–	0.1 ± 0.5 (0.1)	–	2 ± 1.9 (3.1)	4.7 ± 2.4 (19.9)

**Table 2:** The mean ± standard deviation of the mean absolute errors (MAEs) of the trunk forward inclination angle between cloth sensors and skin sensors during the five simulated tasks, with the reference value of skin sensors shown in brackets (*n* = 12).

Trunk, Forward Inclination	Simulated Work Tasks				
	Lifting Boxes	Sorting Mails	Wiping Floor	Cleaning Dishwasher	Cleaning Windows
Percentile (°)					
5th	2.8 ± 1.9 (2.4)	2.6 ± 1.4 (5.6)	2.5 ± 2 (20.5)	3.3 ± 4.1 (−3.8)	3.6 ± 3.3 (−8.4)
10th	2.9 ± 1.9 (5.8)	2.7 ± 1.5 (7.7)	2.6 ± 1.9 (25.2)	3.2 ± 4.8 (−0.7)	4.1 ± 3.3 (−6.1)
50th	3.2 ± 2.7 (23.6)	2.7 ± 1.7 (14.4)	3.1 ± 2.5 (41.9)	3.7 ± 3.9 (20.4)	3.7 ± 3.2 (3.1)
90th	6.8 ± 7.2 (63.8)	2.7 ± 1.7 (20.7)	3.9 ± 3.7 (53.6)	5.6 ± 6.7 (64)	3.9 ± 3.7 (16.6)
95th	6.8 ± 7.2 (67.3)	2.5 ± 1.9 (22.4)	4.1 ± 4.1 (56.3)	5.8 ± 6.4 (70.9)	3.9 ± 3.6 (21.1)
Percentage of time (%)					
<20°	4.8 ± 4.2 (42.5)	10.7 ± 11.4 (78.4)	2.4 ± 2.4 (6.8)	4.5 ± 6.9 (48.6)	4.4 ± 5.9 (93.1)
>30°	4.6 ± 5.3 (43.5)	1.4 ± 2.7 (2.9)	4.8 ± 4.2 (78.1)	3.4 ± 4 (42.6)	1.4 ± 1.7 (2.6)
>45°	4 ± 4.5 (30)	0 ± 0 (0.1)	7 ± 7.1 (36.7)	3.3 ± 4.6 (26.8)	0.2 ± 0.4 (0.5)
>60°	7.2 ± 9.3 (14.8)	0 ± 0.1 (0.1)	6.4 ± 10.9 (8.6)	4.2 ± 7.3 (14)	0 ± 0.1 (0)
>90°	–	–	–	–	–

**Table 3:** The mean ± standard deviation of the mean absolute errors (MAEs) of the dominant upper arm inclination velocity between cloth sensors and skin sensors during the five simulated tasks, with the reference value of skin sensors shown in brackets (*n* = 12).

Dominant Arm, Inclination Velocity	Simulated Work Tasks				
	Lifting Boxes	Sorting Mails	Wiping Floor	Cleaning Dishwasher	Cleaning Windows
Percentile (°/s)					
5th	0.3 ± 0.3 (2.5)	0.4 ± 0.3 (2.1)	0.2 ± 0.1 (2.5)	0.3 ± 0.3 (2.4)	0.4 ± 0.3 (4.3)
10th	0.5 ± 0.6 (6.4)	0.7 ± 0.4 (5.5)	0.4 ± 0.3 (6)	0.5 ± 0.4 (5.8)	0.9 ± 0.6 (10.4)
50th	1.8 ± 1.5 (33.5)	3.3 ± 1.7 (30.1)	1 ± 0.7 (31.8)	1.4 ± 1.2 (32.2)	4.5 ± 3.4 (64.8)
90th	4.1 ± 3.3 (96.3)	10.9 ± 5.5 (90.5)	4.1 ± 4 (91.7)	5.1 ± 4.7 (108.7)	18.8 ± 16.3 (191.6)
95th	5.1 ± 3.8 (121.7)	15.3 ± 7.6 (116.5)	5.5 ± 5.7 (116.2)	9.4 ± 7.3 (142.3)	26.1 ± 24.8 (244.1)

**Table 4:** The mean ± standard deviation of the mean absolute errors (MAEs) of the trunk inclination velocity between cloth sensors and skin sensors during the five simulated tasks, with the reference value of skin sensors shown in brackets (*n* = 12).

Trunk, Forward Inclination Velocity	Simulated Work Tasks				
	Lifting Boxes	Sorting Mails	Wiping Floor	Cleaning Dishwasher	Cleaning Windows
Percentile (°/s)					
5th	0.3 ± 0.2 (2.6)	0.1 ± 0.1 (1)	0.1 ± 0.1 (1.4)	0.2 ± 0.1 (1.6)	0.2 ± 0.2 (1.9)
10th	0.3 ± 0.3 (5)	0.1 ± 0.1 (2)	0.2 ± 0.1 (2.9)	0.3 ± 0.2 (3.2)	0.2 ± 0.2 (4.2)
50th	2 ± 3.1 (27.7)	0.4 ± 0.3 (9.8)	1 ± 0.7 (15.2)	0.9 ± 1.2 (17.6)	1.3 ± 0.7 (22.3)
90th	11 ± 10.7 (115.5)	1.6 ± 1 (28.7)	2.9 ± 2 (48.5)	3.5 ± 3.1 (64.5)	3.3 ± 1.8 (64.2)
95th	13.2 ± 12.1 (154.5)	2.1 ± 1.3 (36.9)	3.5 ± 3 (64)	5.2 ± 5.2 (87.8)	3.2 ± 1.8 (81.8)

## Data Availability

Data presented in the paper are available upon request from the corresponding author L.Y.

## Appendix A

**Table A1:** The mean ± standard deviation of the mean absolute errors (MAEs) of the non-dominant arm inclination angle between cloth sensors and skin sensors during the five simulated tasks, with the reference value of skin sensors shown in brackets (*n* = 12).

Non-Dominant Arm, Inclination	Simulated Work Tasks				
	Lifting Boxes	Sorting Mails	Wiping Floor	Cleaning Dishwasher	Cleaning Windows
Percentile (°)					
5th	1.1 ± 2 (3.8)	1.4 ± 2.5 (3.5)	2.2 ± 1.7 (9.5)	0.9 ± 1 (5.8)	1.2 ± 1.6 (3.3)
10th	1.4 ± 2.2 (5.4)	1.4 ± 2.5 (4.6)	2.4 ± 1.7 (12.6)	0.9 ± 0.6 (8.6)	1.4 ± 2 (4.7)
50th	1.3 ± 1.7 (15.6)	1.7 ± 2.3 (9.7)	2 ± 1.8 (29.8)	1.9 ± 1.1 (28.8)	1.7 ± 2.2 (13.3)
90th	2.4 ± 1.9 (30.6)	2.2 ± 2.6 (19.4)	2.6 ± 2.4 (51.9)	5.5 ± 4.3 (72.9)	2.8 ± 3.5 (37.6)
95th	3 ± 2.1 (35)	2.8 ± 2.9 (24.4)	2.7 ± 2.4 (57.2)	6.5 ± 4.1 (83.2)	5.4 ± 4.3 (50.8)
Percentage of time (%)					
<20°	4.8 ± 3.8 (65.2)	7.8 ± 20.4 (91.5)	4.3 ± 2.5 (28.9)	3.5 ± 2.3 (37.1)	4.7 ± 6.8 (71.8)
>30°	4.2 ± 5.1 (11.7)	0.9 ± 1.6 (2.3)	3.7 ± 3.3 (48)	2.8 ± 2.3 (47.4)	1.4 ± 2.1 (14.1)
>45°	0.6 ± 1.4 (0.9)	0.4 ± 0.8 (1.2)	3.9 ± 4.9 (22.8)	1.5 ± 1.5 (29.7)	1.2 ± 1.5 (7.4)
>60°	0 ± 0 (0)	0.2 ± 0.3 (0.4)	2.1 ± 3.4 (4.7)	2.2 ± 2.1 (17.6)	0.9 ± 1.3 (4.3)
>90°	0 ± 0 (0)	0 ± 0.1 (0)	0 ± 0 (0.1)	1.8 ± 1.8 (3.4)	0.5 ± 0.8 (1.4)

**Table A2:** The mean ± standard deviation of the mean absolute errors (MAEs) of the non-dominant arm inclination velocity between cloth sensors and skin sensors during the five simulated tasks, with the reference value of skin sensors shown in brackets (*n* = 12).

Non-Dominant Arm, Inclination Velocity	Simulated Work Tasks				
	Lifting Boxes	Sorting Mails	Wiping Floor	Cleaning Dishwasher	Cleaning Windows
Percentile (°/s)					
5th	0.3 ± 0.2 (2.7)	0.2 ± 0.1 (1.5)	0.2 ± 0.1 (1.8)	0.2 ± 0.2 (2.3)	0.3 ± 0.2 (2.6)
10th	0.6 ± 0.4 (6.6)	0.3 ± 0.2 (3.5)	0.2 ± 0.1 (4.4)	0.3 ± 0.3 (5.2)	0.4 ± 0.2 (5.8)
50th	2.1 ± 2.1 (34)	1 ± 0.8 (17.4)	0.5 ± 0.5 (23.5)	1.4 ± 1.1 (30.3)	1.6 ± 1.3 (30.9)
90th	4.9 ± 4.7 (97.7)	3.5 ± 2.4 (52.7)	1.9 ± 1.6 (68.6)	5.5 ± 4.6 (106)	3.7 ± 3 (94.7)
95th	6.6 ± 5.4 (124.6)	4.9 ± 3.7 (69.3)	1.9 ± 1.5 (88)	6.6 ± 4 (140.5)	5 ± 4.3 (122.2)

**Table A3:** The mean ± standard deviation of the mean absolute errors (MAEs) of the dominant arm generalized velocity between cloth sensors and skin sensors during the five simulated tasks, with the reference value of skin sensors shown in brackets (*n* = 12).

Dominant Arm, Generalized Velocity	Simulated Work Tasks				
	Lifting Boxes	Sorting Mails	Wiping Floor	Cleaning Dishwasher	Cleaning Windows
Percentile (°/s)					
5th	1.1 ± 0.6 (11.3)	1.5 ± 0.7 (11.6)	0.6 ± 0.4 (11.1)	0.9 ± 0.6 (10.8)	1.8 ± 1.4 (19.4)
10th	1.4 ± 0.7 (17.1)	2.1 ± 0.8 (17.4)	0.9 ± 0.5 (17.3)	1.2 ± 0.9 (16.6)	2.4 ± 2 (31.3)
50th	4.5 ± 4.3 (53)	7 ± 2.9 (53.1)	5.6 ± 3.3 (57.6)	3.8 ± 2.2 (58.5)	15.3 ± 5.1 (124.2)
90th	21.9 ± 28.1 (134.9)	20.5 ± 11.9 (132.2)	39.7 ± 29.5 (179.9)	15.6 ± 18.7 (170.8)	54 ± 30.1 (324.6)
95th	30.7 ± 40.9 (174.7)	25.9 ± 16.1 (164.9)	58.4 ± 49.7 (256.6)	26.5 ± 35.3 (235.7)	71.5 ± 40.2 (398.1)

**Table A4:** The mean ± standard deviation of the mean absolute errors (MAEs) of the non-dominant arm generalized velocity between cloth sensors and skin sensors during the five simulated tasks, with the reference value of skin sensors shown in brackets (*n* = 12).

Non-Dominant arm, Generalized Velocity	Simulated Work Tasks				
	Lifting Boxes	Sorting Mails	Wiping Floor	Cleaning Dishwasher	Cleaning Windows
Percentile (°/s)					
5th	1.1 ± 1.2 (11.9)	0.7 ± 0.4 (6.8)	0.6 ± 0.7 (9.2)	1 ± 0.8 (9.6)	0.9 ± 0.9 (12.3)
10th	1.6 ± 1 (17.9)	0.8 ± 0.6 (10.4)	0.9 ± 0.8 (13.7)	1.2 ± 0.7 (14.7)	1 ± 1.1 (18.3)
50th	3.9 ± 3.8 (55.2)	2.7 ± 2.4 (32.7)	2.3 ± 1.4 (41.1)	3.6 ± 1.5 (53.4)	3.4 ± 1.6 (56.6)
90th	29.7 ± 42.1 (145.3)	12.9 ± 17 (94.1)	12.7 ± 23.2 (104.4)	12.5 ± 8.4 (159.4)	18 ± 23.6 (148.5)
95th	39.1 ± 53.2 (191.9)	12.4 ± 8.6 (123.4)	25.8 ± 49.5 (136.6)	22.9 ± 18.7 (216.4)	27.4 ± 35.3 (189.9)
